# Vaping cessation: how to treat nicotine dependence and tailor the nicotine replacement dose. A narrative review

**DOI:** 10.36416/1806-3756/e20250061

**Published:** 2025-09-22

**Authors:** Stella Regina Martins, Paulo César Rodrigues Pinto Corrêa, Carolina Costa, Márcio Gonçalves de Sousa, Cristiane Almeida Pires Tourinho, Vera Lúcia Gomes Borges

**Affiliations:** 1. Seção de Hipertensão, Tabagismo e Nefrologia, Instituto Dante Pazzanese de Cardiologia, São Paulo (SP) Brasil.; 2. Escola de Medicina, Universidade Federal de Ouro Preto, Ouro Preto (MG) Brasil.; 3. Programa de Pós-Graduação em Saúde Pública, Universidade Federal de Minas Gerais, Belo Horizonte (MG) Brasil.; 4. Instituto de Psiquiatria, Universidade Federal do Rio de Janeiro, Rio de Janeiro (RJ) Brasil.; 5. Ambulatório de Cessação de Tabagismo, Serviço de Pneumologia, Universidade do Estado do Rio de Janeiro, Rio de Janeiro (RJ) Brasil.; 6. Coordenação de Prevenção e Vigilância de Câncer, Divisão de Controle do Tabagismo - DITAB - Instituto Nacional de Câncer - INCA - Rio de Janeiro, Rio de Janeiro (RJ) Brasil.

**Keywords:** Vaping, Cessation Guidance, Electronic nicotine delivery systems, nicotine replacement therapy, E-cigarette, Addiction medicine

## Abstract

Electronic nicotine delivery systems, electronic cigarettes, or vapes have been extensively marketed as a safer alternative to combustible cigarettes and as aids for smoking cessation. However, electronic cigarettes often deliver more potent forms of nicotine, such as nicotine salts and synthetic nicotine, which are masked by appealing aromas and flavors, thereby attracting nonsmoking children and adolescents. On the other hand, adults dependent on freebase nicotine (found in conventional cigarettes) often become addicted to these new forms of nicotine in electronic cigarettes. Dual use is common and poses significant health risks, potentially exceeding those of using either product alone. Dual users experience increased odds of COPD, lung cancer, cardiovascular disease, and stroke. Electronic cigarettes represent a new challenge for global public health and health professionals. There are currently no specific guidelines for vaping cessation treatment. This study sought to provide health professionals with a comprehensive vaping cessation approach, including effective strategies such as behavioral support, nicotine replacement therapy, and the use of nicotine-free medications.

## INTRODUCTION

Electronic cigarettes (ECs), also known as e-cigarettes, e-cigs, or vapes, are types of electronic nicotine delivery systems. ECs consist of a lithium battery, e-liquids, propylene glycol, vegetable glycerin, nicotine (most often), additives, and other substances that are harmful to health. The act of using ECs is known as “vaping,” and EC users do not identify themselves as smokers; rather, they call themselves “vapers.”^1^ ^,^ ^2^

Social media plays a key role in the illegal sale and promotion of ECs, creating a false sense of security with misleading information delivered through influencers and hashtag campaigns sponsored by the tobacco industry. These strategies lead EC users to believe that they are simply inhaling water vapor or a less harmful product.^2^ In reality, ECs contain several toxic and carcinogenic substances, such as heavy metals and different types of additives with attractive aromas and flavors, such as kiwi or peach, which facilitate the absorption of countless harmful substances, as well as new forms of nicotine, which are more addictive and which are not used in combustible cigarettes.^1^

Dual use of combustible cigarettes and ECs is a prevalent behavior associated with a significantly increased risk of cardiovascular, cerebrovascular, and pulmonary diseases such as COPD and lung cancer, thereby contributing to elevated rates of premature mortality.^3^ ^-^ ^5^

In 2021, The WHO estimated that there were 82 million EC users worldwide, EC use being more prevalent among children in the 13- to 15-year age bracket than among people in older age groups, as well as being more prevalent among boys than among girls.^6^ Therefore, ECs are a new and major challenge for global public health. 

In the present study, we sought to investigate issues such as the wide variety of electronic nicotine delivery systems; the breadth of nicotine products currently available on the market; and the patterns of EC use. We also examined approaches to vaping cessation; pharmacological treatment with or without nicotine replacement; and how to tailor the nicotine replacement dose. 

## PHYSICAL, PSYCHOLOGICAL, AND BEHAVIORAL ASPECTS OF ADDICTION

In childhood and adolescence, the brain is still developing; nicotine affects areas responsible for memory, feelings, thoughts, and decision-making processes and can cause serious damage to mental and physical health, leading to nicotine addiction.^7^ Addiction is characterized by a failure to control drug use. In other words, physical, psychological, and behavioral disorders occur in the absence of the drug, including insomnia, cramps, dizziness, anxiety, irritability, craving, and agitation.^7^

The psychological aspect of dependence encompasses the meaning or function that smoking has for the user. The device itself can be used in order to deal with feelings and emotions, as well as for relaxation or coping with stressful situations.^7^ There is also the behavioral component of addiction: several associations of behaviors linked to rituals and pairing of habits via implicit memory are obstacles to quitting smoking.^7^

ECs have a pleasant smell and taste, and the packaging has beautiful colors, evoking affective memories of smells, tastes, and colors; in addition, associations of EC use with pleasurable social experiences can lead to cognitive distortions that cloud the understanding of the risks of EC use.^8^ ^,^ ^9^

## TREATMENT OF NICOTINE ADDICTION

### Motivational interviewing and cognitive behavioral therapy

Addiction to ECs and dual use are emerging public health challenges that require evidence-based and tailored therapeutic approaches. Although strategies used for conventional smoking cessation are applicable, EC addiction management requires adaptations to address unique patterns of nicotine use and a younger population of users. 

Communication skills are essential for establishing a therapeutic relationship and encouraging patient engagement in treatment. Miller and Rollnick’s motivational interviewing is widely recognized as an important therapeutic approach in clinical settings. It is a patient-centered approach that incorporates core principles such as asking open-ended questions, active listening, providing affirmation (praise, recognition, and understanding), and summarizing the contents mentioned back to the patient. The main goal is to elicit the person’s own reasons for change in an atmosphere of empathy and acceptance.^10^ It can be combined with other therapeutic methods such as cognitive behavioral therapy (CBT).^11^

In CBT for addictive disorders, five critical components of a therapy session are structure (including mood assessment, setting the agenda, action planning, and gathering patient feedback); collaboration; case conceptualization; psychoeducation; and the use of standardized techniques. By collaboratively exploring emotions, behaviors, thoughts, and beliefs with the patient, a more feasible and effective plan for behavior change can be developed. Addiction-related thoughts and beliefs are pivotal for the addiction process. Other specific cognitive processes in addiction include self-efficacy, positive outcome expectancies, negative outcome expectancies, and permissive beliefs (i.e., “I will just use it today.”) Instrumental thoughts, which guide the logistics of addictive behaviors (such as how to obtain the substance), also need to be assessed.^12^

Some cognitive behavioral techniques have shown promise in the treatment of drug addiction and, consequently, can be useful for the nonpharmacological treatment of EC addiction. For example, cognitive restructuring consists of helping patients question their distorted thoughts and beliefs on the basis of more realistic evidence, including evidence on the role that substance use plays in the physical and mental health of users. When these cognitive distortions are altered and made more flexible, it becomes possible to change the emotional state and, consequently, the behavior of users. The development of coping and problem-solving strategies can also improve the response to treatment.^13^

Behavioral support plays a pivotal role in the treatment of EC addiction. Counseling programs assist users in developing strategies for cessation and have been shown to enhance the efficacy of pharmacological and behavioral interventions.^14^ The combined use of psychological support and medication can improve outcomes and adherence to treatment protocols. 

Although there is limited evidence for structured therapeutic interventions for EC addiction, tools such as smartphone applications are currently being developed and investigated. Although further studies are needed to evaluate their quality, content, user acceptance, and effectiveness, these tools can support EC cessation through text messages that target cognitive aspects and promote behavior change. 

In a randomized controlled trial for vaping cessation, 2,588 participants in the 18- to 24-year age bracket were recruited for a fully automated text message intervention delivering social support, as well as cognitive and behavioral coping skills training, with the control arm receiving assessment only. The content included self-efficacy exercises, coping strategies, information about the risks of vaping, the benefits of quitting, how to cut down to quit, and distraction and substitution tips. Thirty-day abstinence rates after 7 months of follow-up were 24.1% (95% CI, 21.8-26.5) in the intervention group and 18.6% (95% CI, 16.7-20.8) in the control group (OR, 1.39; 95% CI, 1.15-1.68; p < 0.001).^15^ However, in a recent systematic review of interventions for quitting vaping, evidence for text message-based interventions were considered of low certainty for vaping cessation in comparison with control in participants in the 13- to 24-year age bracket (two studies, 4,091 participants).^16^

### How to decide when pharmacological treatment is necessary

The decision to initiate pharmacological treatment for nicotine dependence, including vaping, should be based on a comprehensive assessment of patient history, prior cessation attempts, level of nicotine dependence, and severity of withdrawal symptoms.^13^ Given the amplified health risks of dual EC and combustible cigarette use, complete cessation of all tobacco and nicotine products is the major therapeutic goal.^17^

Nicotine replacement therapy (NRT), bupropion, varenicline, and cytisine (also known as cytisinicline), when used in combination with CBT, can reduce withdrawal symptoms and cravings, aiding in overcoming nicotine dependence, regardless of the delivery method.^13^ Extrapolating from the established success of evidence-based pharmacological treatment for smoking cessation, similar strategies are reasonably applicable to EC users, even as specific guidelines evolve. 

Early studies support this approach, with varenicline demonstrating a 40% continuous abstinence rate in comparison with 20% with placebo^18^ and cytisinicline showing efficacy for EC cessation (31.8% vs 15.1% with placebo), suggesting potential pharmacological options for adults seeking to quit vaping.^19^

### NRT for vapers

NRT is available in a variety of ways to suit different needs and levels of addiction. There is fast-acting nicotine (e.g., 2 or 4 mg gums or lozenges, sprays, and inhalers), which can be used in order to manage sudden cravings. Another form of NRT is slow-acting nicotine patches, which provide constant support throughout the day (e.g., 21, 14, and 7 mg transdermal patches).^20^

The maximum dose of NRT depends on the form used. For patches, it is usually not recommended to exceed a dose of 42 mg/day, but individual assessments are necessary. For gums and lozenges, the dose should be limited to approximately 15 to 20 gums or lozenges per day. Higher doses are recommended for heavier nicotine users.^20^

For early-stage treatment regimens, it is recommended to start with a higher dose (e.g., 21 mg patches). As patients wean themselves from ECs (i.e., in the maintenance phase of treatment), gradual reductions in NRT are recommended. After 6 to 12 weeks, lower doses should be prescribed (e.g., 14 mg patches and then 7 mg patches).^20^

In the weaning phase, a gradual reduction in dose over 8 to 12 weeks is recommended. Patients should be supported throughout the treatment with behavioral counseling to increase efficacy.^20^

Special attention should be paid to nicotine overdose situations, in which symptoms such as nausea, vomiting, increased heart rate, and dizziness may occur. Health care professionals should inform users of these signs of toxicity.^20^

Contraindications such as cardiovascular disease (e.g., recent myocardial infarction and unstable angina) and being pregnant or breastfeeding should be considered by health care professionals during treatment planning.^20^

### How to tailor the nicotine replacement dose for EC users

The wide variety of ECs and liquid nicotine products available on the market has made it challenging for health care professionals to tailor the nicotine replacement dose. 

Clinically relevant measures of EC use include the number of times the device is used/day; the number of puffs/day; the volume of liquid nicotine consumed/day; the concentration and type of liquid nicotine in ECs; EC generation; and EC electrical power (in watts). However, there are several limitations. First, one distinctive characteristic of EC use is automatic use throughout the day-a behavior known as “grazing”-whereas, with tobacco cigarette smoking, there is a clear start and a clear end to smoking each cigarette.^21^ ^,^ ^22^ Second, EC users are not always able to provide correct information on the device characteristics, such as power, nicotine content, and composition.^23^ ^-^ ^28^

Very few trials on vaping cessation have been conducted. To our knowledge, there are currently no evidence-based guidelines regarding pharmacological interventions for vaping cessation. In a review of vaping cessation interventions available to former smokers, the authors investigated ECs as a smoking cessation method.^29^ Given that EC users worldwide are increasingly seeking vaping cessation treatment, a reasonable approach is to use the scientific evidence available for combustible cigarette smoking cessation, including the use of NRT and other first-line medications in combination with CBT. 

The correct use of NRT for EC users is challenging and of paramount importance.^1^ Specialists or clinicians aiding EC users in vaping cessation should collect data on three parameters in order to measure EC use:

- device features-EC type (mod or pod); rechargeable or disposable; brand/brands used; and number of puffs the device delivers
- e-liquid characteristics: type of nicotine (freebase nicotine, nicotine salt, or synthetic nicotine); amount of e-liquid (mg/mL) per refill or per tank; and nicotine concentration (%; mg/mL) per refill
- EC use characteristics: refill frequency or time until next purchase of a disposable pod; number of e-liquids used in the tank; final concentration of nicotine in the e-liquid after filling the tank with more than one e-liquid; and dual use of combustible cigarettes and ECs

If a health care professional chooses to use NRT for vaping cessation, they must calculate the approximate nicotine dose. To that end, the following information is required: nicotine type and concentration (in % or mg/mL); tank/device capacity (in mL); and the time it takes to consume all of the e-liquid. Brazilian legislation determines that the maximum nicotine concentration in firsthand smoke must be 1 mg per cigarette.^30^ The idea is to use NRT to replace the amount of nicotine consumed per day. 

### Hypothetical clinical case

**Figure 1:**
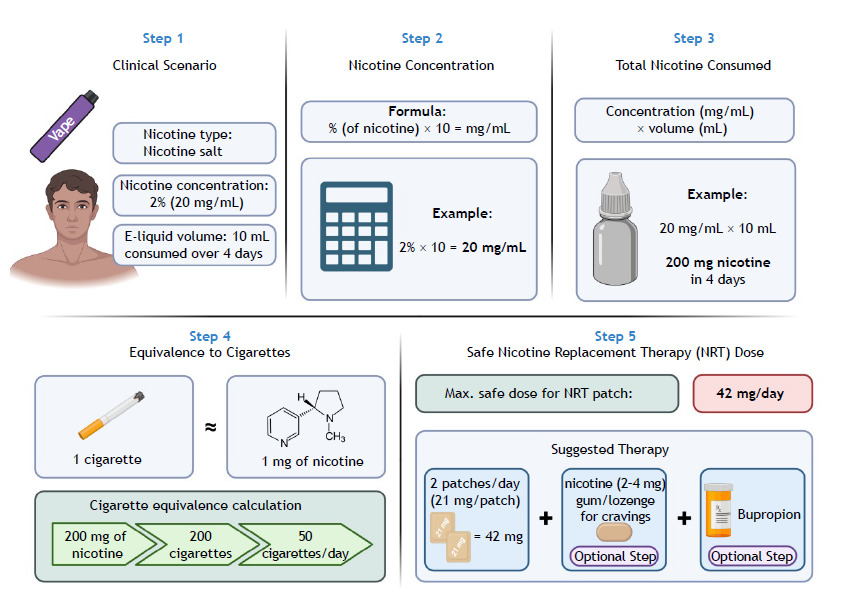
Example of approximate calculation of nicotine equivalence.

**Figure 2:**
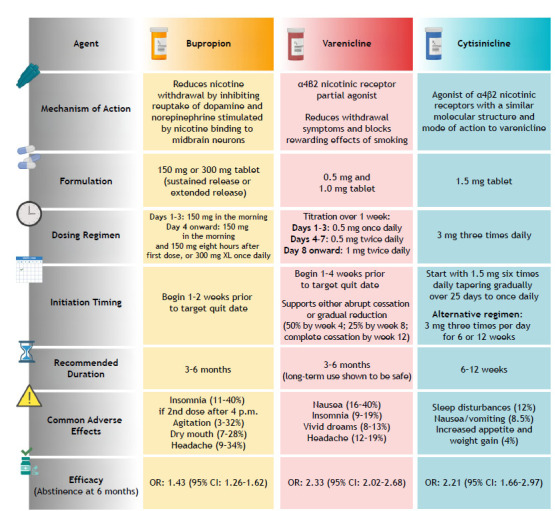
Non-nicotine pharmacological treatment for smoking cessation and smoking abstinence at 6 months. Adapted from Barua et al. and Lindson et al.^17^ ^,^ ^34^

A 20-year-old male seeks vaping cessation treatment. He reports using the following: nicotine salts at a concentration of 2%; an EC the total capacity of which is 10 mL of e-liquid; and 10 mL of e-liquid in 4 days. How can the health professional aiding in vaping cessation calculate the approximate amount of nicotine consumed, the equivalence between EC use and combustible cigarette use, and a safe nicotine replacement dose (Figure 1)? Among pharmacological treatments for smoking cessation, bupropion, varenicline, and cytisinicline stand out (Figure 2). 

Bupropion, an antagonist of nicotinic receptors and dopamine reuptake inhibitor, helps alleviate withdrawal symptoms and reduces cravings for ECs. This pharmacological approach can be combined with behavioral support to optimize treatment outcomes.^17^ Bupropion reduces seizure threshold and should not be used in patients who are at an increased risk of seizures.^17^

Varenicline, a partial agonist of the α4β2 nicotinic receptors, has proven efficacy in aiding EC cessation by reducing cravings and the reinforcing effects associated with nicotine consumption. Studies suggest that varenicline is preferable as a cessation strategy because of its safety and efficacy profile.^31^ ^,^ ^32^

In a randomized clinical trial, the efficacy of varenicline (1 mg twice daily for 12 weeks) combined with counseling was evaluated in daily EC users seeking to quit. The results demonstrated significantly higher continuous abstinence rates with varenicline in comparison with placebo: 40% vs. 20% at weeks 4 to 12 and 34.3% vs. 17.2% at weeks 4 to 24. Although these findings reinforce the effectiveness of varenicline, they also indicate a low incidence of serious adverse events unrelated to the medication.^18^

In a recently published randomized clinical trial, the effectiveness of varenicline for nicotine vaping cessation was evaluated among individuals in the 16- to 25-year age bracket who vaped daily or near daily but did not regularly smoke tobacco. Participants were assigned to three groups: 12 weeks of double-blind varenicline with behavioral counseling and text messaging support (n = 88), identical placebo with counseling and support (n = 87), or referral to support only (enhanced usual care, n = 86). The primary outcome was biochemically verified continuous vaping abstinence during the last 4 weeks of treatment. Results showed that abstinence was 51% with varenicline vs. 14% with placebo during weeks 9-12 and 28% vs. 7% during weeks 9-24, indicating a significant benefit of varenicline. Varenicline also outperformed enhanced usual care, with higher abstinence rates. The authors of the study concluded that varenicline significantly improved vaping cessation in that population.^33^

Cytisinicline is an alkaloid that occurs naturally in several plant genera. Its molecular structure has some similarity to that of nicotine and varenicline, and it has the same pharmacological effects: it is a partial agonist of brain nicotinic acetylcholine receptors. It decreases the urge to use tobacco and reduces the severity of nicotine withdrawal symptoms, while also reducing the reward experience of using tobacco.^34^

## FINAL CONSIDERATIONS

The treatment of EC dependence requires an integrated approach combining behavioral interventions and pharmacotherapy, with the ultimate goal being complete nicotine cessation. 

The present study provides health care professionals with a comprehensive cessation framework, including strategies for behavioral support; safe and effective use of NRT; and non-nicotine pharmacological alternatives. A key contribution of our study is the methodology for calculating nicotine equivalence, which facilitates accurate NRT dosing and supports clinical decision-making in the management of nicotine addiction. Further studies are needed to strengthen the evidence base and inform best practices in the treatment of EC dependence. 

## References

[1] Soule E, Bansal-Travers M, Grana R, McIntosh S, Price S, Unger JB (2023). Electronic cigarette use intensity measurement challenges and regulatory implications. Tob Control.

[2] Barufaldi LA, Guerra RL, Albuquerque RCR, Nascimento AD, Chança RD, Souza MC (2021). Risk of initiation to smoking with the use of electronic cigarettes systematic review and meta-analysis [Article in Portuguese]. Cien Saude. Colet.

[3] Ashraf MT, Shaikh A, Khan MKS, Uddin N, Kashif MAB, Rizvi SHA (2023). Association between e-cigarette use and myocardial infarction a systematic review and meta-analysis. Egypt Heart J.

[4] Parekh T, Pemmasani S, Desai R (2020). Risk of Stroke With E-Cigarette and Combustible Cigarette Use in Young Adults. Am J Prev Med.

[5] Song B, Li H, Zhang H, Jiao L, Wu S (2024). Impact of electronic cigarette usage on the onset of respiratory symptoms and COPD among Chinese adults. Sci Rep.

[6] World Health Organization [homepage on the Internet] (c2025). https://www.who.int/publications/i/item/9789240032095.

[7] Brasil. Ministério da Saúde. Instituto Nacional do Câncer (INCA) Cigarros eletrônicos: o que sabemos.

[8] Lima e Silva J.Rebessi IP.Antonelli-Ponti M (2024). O processo de ensino-aprendizagem e a regulação emocional: encontros entre a terapia cognitivo-comportamental e a neuropsicologia.

[9] Knapp P, Beck AT (2008). Cognitive therapy foundations, conceptual models, applications and research [Article in Portuguese]. Braz. J Psychiatry.

[10] Bischof G, Bischof A, Rumpf HJ (2021). Motivational Interviewing An Evidence-Based Approach for Use in Medical Practice. Dtsch Arztebl Int.

[11] Magill M, Ray L, Kiluk B, Hoadley A, Bernstein M, Tonigan JS (2019). A meta-analysis of cognitive-behavioral therapy for alcohol or other drug use disorders Treatment efficacy by contrast condition. J Consult Clin Psychol.

[12] Liese B, Beck A (2022). Cognitive-Behavioral Therapy of Addictive Disorders.

[13] Padesky CA (2020). Collaborative case conceptualization Client knows best. Cogn Behav Pract.

[14] Shields PG, Bierut L, Arenberg D, Balis D, Cinciripini PM, Davis J (2023). Smoking Cessation, Version 3 2022, NCCN Clinical Practice Guidelines in Oncology. J Natl Compr Canc Netw.

[15] Graham AL, Amato MS, Cha S, Jacobs MA, Bottcher MM, Papandonatos GD (2021). Effectiveness of a Vaping Cessation Text Message Program Among Young Adult e-Cigarette Users A Randomized Clinical Trial. JAMA Intern Med.

[16] Butler AR, Lindson N, Livingstone-Banks J, Notley C, Turner T, Rigotti NA (2024). Interventions for quitting vaping. Cochrane Database Syst Rev.

[17] Barua RS, Rigotti NA, Benowitz NL, Cummings KM, Jazayeri MA, Morris PB (2018). 2018 ACC Expert Consensus Decision Pathway on Tobacco Cessation Treatment A Report of the American College of Cardiology Task Force on Clinical Expert Consensus Documents. J Am Coll Cardiol.

[18] Caponnetto P, Campagna D, Ahluwalia JS, Russell C, Maglia M, Riela PM (2023). Varenicline and counseling for vaping cessation a double-blind, randomized, parallel-group, placebo-controlled trial. BMC Med.

[19] Rigotti NA, Benowitz NL, Prochaska JJ, Cain DF, Ball J, Clarke A (2024). Cytisinicline for Vaping Cessation in Adults Using Nicotine E-Cigarettes The ORCA-V1 Randomized Clinical Trial. JAMA Int Med.

[20] Sandhu A, Hosseini SA, Saadabadi A (2025). Nicotine Replacement Therapy.

[21] Ng G, Attwells S, Zawertailo L (2022). The development and validation of an electronic nicotine delivery system (ENDS) image cue stimulus set. Drug Alcohol Depend.

[22] Fenton E, Robertson L, Hoek J (2023). Ethics and ENDS. Tob Control.

[23] Inter-university Consortium for Political and Social Research (2024). Population Assessment of Tobacco and Health (PATH) Study.

[24] Kim H, Davis AH, Dohack JL, Clark PI (2017). E-Cigarettes Use Behavior and Experience of Adults Qualitative Research Findings to Inform E-Cigarette Use Measure Development. Nicot Tob Res.

[25] Raymond BH, Collette-Merrill K, Harrison RG, Jarvis S, Rasmussen RJ (2018). The Nicotine Content of a Sample of E-cigarette Liquid Manufactured in the United States. J Addict Med.

[26] Rudy AK, Leventhal AM, Goldenson NI, Eissenberg T (2017). Assessing electronic cigarette effects and regulatory impact Challenges with user self-reported device power. Drug Alcohol Depend.

[27] Wagener TL, Floyd EL, Stepanov I, Driskill LM, Frank SG, Meier E (2017). Have combustible cigarettes met their match The nicotine delivery profiles and harmful constituent exposures of second-generation and third-generation electronic cigarette users. Tob Control.

[28] CASEL ENDS Measures Subcommittee (2020). Recommendations for ends core measures for TCORS studies..

[29] Huerne K, Eisenberg MJ (2023). Vaping-Cessation Interventions in Former Smokers. The Can J Cardiol.

[30] Brasil. Ministério da Saúde. Agência Nacional de Vigilancia Sanitária (ANVISA) (c2012). https://bvsms.saude.gov.br/bvs/saudelegis/anvisa/2012/rdc0014_15_03_2012.html.

[31] Grubb LK, COMMITTEE ON ADOLESCENCE (2020). Barrier Protection Use by Adolescents During Sexual Activity. Pediatrics.

[32] Tuisku A, Rahkola M, Nieminen P, Toljamo T (2024). Electronic Cigarettes vs Varenicline for Smoking Cessation in Adults A Randomized Clinical Trial. JAMA Int Med.

[33] Evins AE, Cather C, Reeder HT, Evohr B, Potter K, Pachas GN (2025). Varenicline for Youth Nicotine Vaping Cessation A Randomized Clinical Trial. JAMA.

[34] Lindson N, Theodoulou A, Ordóñez-Mena JM, Fanshawe TR, Sutton AJ, Livingstone-Banks J (2023). Pharmacological and electronic cigarette interventions for smoking cessation in adults component network meta-analyses. Cochrane Database Syst Rev.

